# Effect of Weekend Admission on Hip Fracture Mortality

**DOI:** 10.31486/toj.24.0017

**Published:** 2025

**Authors:** Nicholas L. Newcomb, Marlena Urvater, Ian E. Doig, Michael Mullen, Cameron M. Cooke

**Affiliations:** ^1^ Department of Orthopaedics, University of New Mexico, Albuquerque, NM; ^2^ The University of Queensland Medical School, Ochsner Clinical School, New Orleans, LA; ^3^ Department of Emergency Medicine, Cape Fear Valley Health, Fayetteville, NC; ^4^ Department of Orthopaedics, Princess Alexandra Hospital, Woolloongabba, Queensland, Australia

**Keywords:** *Hip fractures*, *hospitalization*, *operative time*, *treatment delay*, *weekend effect*

## Abstract

**Background:**

Weekend vs weekday hospital admission has been associated with poorer mortality rates for many conditions. Studies evaluating weekend admission for hip fractures have resulted in contradictory conclusions regarding outcomes.

**Methods:**

We conducted a retrospective analysis of all patients who underwent surgery for a fragility hip fracture at a quaternary level teaching hospital during a 6-year period. A total of 1,164 patients were included: 796 weekday admissions (Monday through Friday) vs 368 weekend admissions (Saturday and Sunday). Patients were subdivided based on surgeon experience level (473 consultants vs 690 nonconsultants). Statistical tests included chi-square tests and logistic regression. Demographic data included age, sex, prior hip fracture, fracture type, operation, and American Society of Anesthesiologists grade. The primary outcome was 1-year mortality. Secondary outcomes were acute mortality (<24 hours), subacute mortality (1 to 30 days), change in mobility from baseline at 1 year, preoperative delay (>48 hours), and surgical duration.

**Results:**

The weekend admission cohort had a higher 1-year mortality rate than the weekday admission cohort (30.4% vs 23.2%; *P*=0.029), while subacute mortality trended toward significance (*P*=0.083). No significant difference was seen in acute mortality (*P*=0.5). Hemiarthroplasty was associated with increased mortality at 12 months (*P*=0.012) compared to the other operative interventions. The median duration of surgery was lower in the weekend cohort vs the weekday cohort (1.15 hours [69 minutes] vs 1.23 hours [73.8 minutes]; *P*<0.001). Consultants performed surgeries 16.2 minutes faster than nonconsultants (*P*<0.001) and trended toward a lower 1-year mortality rate (22.1% vs 27.9%; *P*=0.058). No significant difference was seen in mobility change at 1 year in both the consultant vs nonconsultant analysis (*P*>0.9) and in the weekday vs weekend analysis (*P*>0.12).

**Conclusion:**

A significantly increased 1-year mortality rate and a shorter surgical duration were observed among patients admitted on the weekends.

## INTRODUCTION

Globally, more than 1 million people sustain hip fractures yearly, with the worldwide prevalence expected to increase from 1.6 million in 2000 to between 4.5 and 6.3 million by 2050.^1-3^ This increasing prevalence, driven by an aging population, resulted in 50,900 hospitalizations between 2015 and 2016 in Australia alone.^4^ Even with advances in care, mortality rates remain high at approximately 30% within 1 year of fracture.^5^ Postoperative challenges include diminished mobility and ability to perform activities of daily living.^6,7^ The severe impact on patients underscores the potential for any improvements in operation efficacy to have a meaningful effect on patient outcomes.

Weekday vs weekend hospital admission has long been theorized to affect patient outcomes. This phenomenon has been termed the “weekend effect,” and multiple conditions have been studied for its impact.^8-11^ Notably, weekend vs weekday hospital admission has been associated with poorer mortality rates for aortic aneurysms, pulmonary embolisms, and stroke.^12,13^ The correlation between hip fractures and the weekend effect has been less clear because of conflicting evidence.

Multiple analyses have found that factors known to affect hip fracture outcomes—including age, sex, American Society of Anesthesiologists (ASA) grade, preoperative delay, preoperative mobility, and fracture type—are associated with increased mortality rates.^14-18^ Lower surgeon experience level has also been associated with poorer surgical outcomes.^19^ However, studies have reported conflicting evidence regarding an association between weekend admissions and mortality.^14,17,20-24^ Potential causes for a weekend effect on hip fracture surgical outcomes are unclear and require further investigation. One factor that has not been robustly studied is the variation in surgeon experience level between weekday and weekend admissions. Likewise, many databases only tracked acute mortality for 30 days. A systematic review by Downey et al found that only 1 hip fracture registry of 8 followed mortality up to 1 year after surgery.^25^ Thus, increased length of monitoring is also indicated to ensure poor outcomes are not missed.

This study examined the relationships between weekend admission, surgeon experience level, and outcomes up to the 1-year time point for patients who underwent operative intervention for hip fracture. We hypothesized that weekend admission would be associated with negative outcomes.

## METHODS

Ethical approval was provided by the Queensland Metro South Human Research Ethics Committee as a negligible risk retrospective study. We then conducted a retrospective analysis of all patients who underwent surgery for a fragility hip fracture at a quaternary level teaching hospital between January 2011 and July 2018. Fragility hip fractures were defined as femoral neck (displaced and nondisplaced intracapsular), intertrochanteric, and subtrochanteric fractures caused by low-energy trauma, such as falls from a standing height. Data were collected from a deidentified hip fracture database maintained by the hospital orthopedics department under institutional review board approval. This database included information on all patients with fragility hip fractures, with 1-year follow-up collected from clinic evaluations and telephone communication with patients. Patients who did not undergo surgery or who were transferred from other facilities were excluded because they had no hospital admission time point. Patients who died after admission but prior to a planned surgery were included in the analysis of mortality relative to the hospital admission time point only.

Subjects were stratified by hospital admission time point—weekday admission (Monday through Friday) vs weekend admission (Saturday and Sunday)—and by surgeon experience level—consultant vs nonconsultant. Consultants are attending-level surgeons (senior medical officers and specialists). Nonconsultants are surgeons in training such as fellows, principal house officers, and registrars (residents).

The primary outcome was mortality up to 1 year after surgery. Secondary outcomes were acute mortality (<24 hours), subacute mortality (1 to 30 days), change in mobility from baseline at 1 year, preoperative delay (measured as a categorical variable ≤48 or >48 hours postadmission), and surgical duration. Baseline mobility was determined using the Parker Mobility Score (scored from 1 to 9) that assesses the ability to move around the house, leave the house, and go shopping.^26^ Higher scores represent better mobility. Demographic variables were age, sex, previous hip fracture, fracture type, and ASA grade. Categorical variables are presented as counts and percentages. Continuous variables are presented using medians and interquartile ranges. Differences between groups were determined using chi-square or Mann-Whitney tests. Statistical analysis was performed by Queensland Facility for Advanced Bioinformatics. The relationships between outcomes and surgeon experience level and between outcomes and hospital admission time point were assessed using chi-square or Mann-Whitney tests. Analysis of the relationship between each outcome and hospital admission time point was performed using logistic regression for binary categorical outcomes. The relationship between each outcome and surgeon experience level was also analyzed using logistic regression. Confounding factors were assessed for significant relationships with hospital admission time point, surgeon experience level, operation type, and outcomes of interest using logistic regression. Significance was determined by *P*<0.05.

## RESULTS

A total of 1,164 patients were included in the analysis, with 796 in the weekday admission cohort vs 368 in the weekend admission cohort (Table 1). The population had a median age of 83 years (range, 58 to 102 years; *P*=0.5); 792 (68.0%) were females and 372 (32.0%) were males (*P*=0.5). One hundred twenty-one patients had had previous hip fractures, while 1,024 had first-time fractures (19 patients unknown) (*P*=0.2). No significant differences were found for fracture type, operation type, or ASA grade between the patients admitted on weekdays vs weekends.

**Table 1.** Patient Demographics Overall and by Hospital Admission Time Point

		Hospital Admission Time Point		
Variable	All Patients, n=1,164	Weekday,^a^ n=796	Weekend,^b^ n=368	*P* Value
Age, years				0.1
≤84	683 (58.7)	480 (60.3)	203 (55.2)	
≥85	481 (41.3)	316 (39.7)	165 (44.8)	
Age, years, median [IQR]	83 [73, 88]	82 [73, 88]	83 [74, 87]	0.5
Sex				0.5
Female	792 (68.0)	537 (67.5)	255 (69.3)	
Male	372 (32.0)	259 (32.5)	113 (30.7)	
Prior hip fracture				0.2
No	1,024 (89.4)	698 (88.6)	326 (91.3)	
Yes	121 (10.6)	90 (11.4)	31 (8.7)	
Unknown	19	8	11	
Fracture type				>0.9
Displaced intracapsular	405 (34.8)	273 (34.3)	132 (35.9)	
Nondisplaced intracapsular	513 (44.1)	351 (44.2)	162 (44.0)	
Intertrochanteric	166 (14.3)	115 (14.5)	51 (13.9)	
Subtrochanteric	79 (6.8)	56 (7.0)	23 (6.3)	
Unknown	1	1	0	
Operation type				0.8
Cannulated screws	42 (3.6)	27 (3.4)	15 (4.1)	
Dynamic hip screws	87 (7.5)	58 (7.3)	29 (7.9)	
Hemiarthroplasty	368 (31.6)	258 (32.4)	110 (29.9)	
Intramedullary nail	531 (45.6)	365 (45.9)	166 (45.1)	
Total hip arthroplasty	136 (11.7)	88 (11.1)	48 (13.0)	
ASA grade				0.2
<3	206 (17.8)	148 (18.7)	58 (16)	
≥3	948 (82.2)	642 (81.3)	306 (84)	
Unknown	10	6	4	
ASA grade, median [IQR]	3.00 [3.00, 3.00]	3.00 [3.00, 3.00]	3.00 [3.00, 3.00]	0.1

^a^Weekday admissions occurred on Monday through Friday.

^b^Weekend admissions occurred on Saturday and Sunday.

Notes: Data are presented as n (%) unless otherwise indicated. *P* values for variables reported as n (%) were determined with the Pearson chi-square test; *P* values for variables reported as median [IQR] were determined with the Mann-Whitney test.

ASA, American Society of Anesthesiologists; IQR, interquartile range.

### Surgeon Experience Level

Surgeon experience level included 473 consultant level, 690 nonconsultant level, and 1 unknown (Table 2). Two significant relationships were found in the surgeon experience level analysis. Consultants were more likely to operate on displaced intracapsular fractures, while nonconsultants were more likely to operate on intertrochanteric and nondisplaced intracapsular fractures (*P*<0.001). Consultants performed 54.3% (220/405) of displaced intracapsular fracture surgeries despite only performing 40.7% (473/1,163) of total surgeries. Comparatively, nonconsultants handled the majority of intertrochanteric (63.3%; 105/166), nondisplaced intracapsular (68.8%; 352/512), and subtrochanteric (59.5%; 47/79) fractures.

**Table 2.** Patient Demographics Overall and by Surgeon Experience Level

		Surgeon Experience Level		
Variable	All Patients, n=1,163^a^	Nonconsultant,^b^ n=690^a^	Consultant,^c^ n=473^a^	*P* Value
Age, years				0.2
≤84	682 (58.6)	393 (57.0)	289 (61.1)	
≥85	481 (41.4)	297 (43.0)	184 (38.9)	
Age, years, median [IQR]	83 [73, 88]	83 [73, 88]	82 [74, 87]	0.7
Sex				0.6
Female	791 (68.0)	473 (68.6)	318 (67.2)	
Male	372 (32.0)	217 (31.4)	155 (32.8)	
Prior hip fracture				0.6
No	1,023 (89.4)	601 (89.0)	422 (90.0)	
Yes	121 (10.6)	74 (11.0)	47 (10.0)	
Unknown	19	15	4	
Fracture type				<0.001
Displaced intracapsular	405 (34.9)	185 (26.9)	220 (46.5)	
Nondisplaced intracapsular	512 (44.1)	352 (51.1)	160 (33.8)	
Intertrochanteric	166 (14.3)	105 (15.2)	61 (12.9)	
Subtrochanteric	79 (6.8)	47 (6.8)	32 (6.8)	
Unknown	1	1	0	
Operation type				<0.001
Cannulated screws	42 (3.6)	33 (4.8)	9 (1.9)	
Dynamic hip screws	87 (7.5)	54 (7.8)	33 (7.0)	
Hemiarthroplasty	368 (31.6)	196 (28.4)	172 (36.4)	
Intramedullary nail	530 (45.6)	363 (52.6)	167 (35.3)	
Total hip arthroplasty	136 (11.7)	44 (6.4)	92 (19.5)	
ASA grade				0.081
<3	206 (17.9)	133 (19.5)	73 (15.5)	
≥3	947 (82.1)	549 (80.5)	398 (84.5)	
Unknown	10	8	2	
ASA grade, median [IQR]	3.00 [3.00, 3.00]	3.00 [3.00, 3.00]	3.00 [3.00, 3.00]	0.2
Hospital admission time point				0.2
Weekday	795 (68.4)	482 (69.9)	313 (66.2)	
Weekend	368 (31.6)	208 (30.1)	160 (33.8)	

^a^Surgeon experience level was not available for 1 patient.

^b^Nonconsultants are surgeons still in training such as fellows, principal house officers, and registrars (residents).

^c^Consultants are attending-level surgeons (senior medical officers and specialists).

Notes: Data are presented as n (%) unless otherwise indicated. *P* values for variables reported as n (%) were determined with the Pearson chi-square test; *P* values for variables reported as median [IQR] were determined with the Mann-Whitney test.

ASA, American Society of Anesthesiologists; IQR, interquartile range.

Consultants were more likely to perform hemiarthroplasties and total hip arthroplasties, while nonconsultants were more likely to perform cannulated screw, dynamic hip screw, and intramedullary nail surgeries (*P*<0.001). We found no relationship between hospital admission time point and surgeon experience level (*P*=0.2).

### Outcomes Analysis by Hospital Admission Time Point

Weekend admission was associated with a 30.4% 1-year mortality rate compared to 23.2% for weekday admission (*P*=0.029) (Table 3). No significant difference was seen between the admission time point cohorts in acute mortality (<24 hours) (*P*=0.5), but the weekend cohort had a higher subacute mortality (1 to 30 days) rate than the weekday cohort (9.9% vs 6.7%, respectively), and the difference trended toward significance (*P*=0.083). The median duration of weekend surgeries was less than the median duration of weekday surgeries (1.15 hours [69 minutes] vs 1.23 hours [73.8 minutes]; *P*≤0.001). Changes in Parker Mobility Score from baseline to the 1-year time point were not significant between the weekday and weekend admission cohorts (*P*=0.12).

**Table 3.** Outcomes by Hospital Admission Time Point

	Hospital Admission Time Point		
Outcome	Weekday,^a^ n=796	Weekend,^b^ n=368	*P* Value
Mortality at 1 year			0.029
No	436 (76.8)	176 (69.6)	
Yes	132 (23.2)	77 (30.4)	
Unknown	228	115	
Acute mortality (<24 hours)			0.5
No	771 (97.0)	354 (96.2)	
Yes	24 (3.0)	14 (3.8)	
Unknown	1	0	
Subacute mortality (1 to 30 days)			0.083
No	603 (93.3)	274 (90.1)	
Yes	43 (6.7)	30 (9.9)	
Unknown	150	64	
Mobility at 1 year			0.13
Improved	62 (14.3)	17 (9.7)	
No change	142 (32.6)	51 (29.0)	
Declined	231 (53.1)	108 (61.4)	
Unknown	361	192	
Change in mobility at 1 year, median [IQR]	–1.00 [–3.00, 0.00]	–1.00 [–3.00, 0.00]	0.12
Preoperative delay (>48 hours)			0.9
No	656 (82.4)	302 (82.1)	
Yes	140 (17.6)	66 (17.9)	
Surgical duration, hours, median [IQR]	1.23 [0.95, 1.60]	1.15 [0.85, 1.46]	<0.001
Unknown	10	5	

^a^Weekday admissions occurred on Monday through Friday.

^b^Weekend admissions occurred on Saturday and Sunday.

Notes: Data are presented as n (%) unless otherwise indicated. Mortality data at 1 year were available for 70.5% (n=821) of patients, while mobility data were available for 52.5% (n=611) of patients. *P* values for variables reported as n (%) were determined with the Pearson chi-square test; *P* values for variables reported as median [IQR] were determined with the Mann-Whitney test.

IQR, interquartile range.

### Outcomes Analysis by Surgeon Experience Level

Analysis of outcomes based on surgeon experience level (Table 4) showed that higher surgeon experience level was associated with decreased duration of surgery, with a median duration of 1.03 hours (61.8 minutes) for consultants vs 1.3 hours (78 minutes) for nonconsultants (*P*<0.001). Additionally, delayed surgeries (>48 hours from admission) were more frequently performed by consultants, with 20.9% of delayed cases performed by consultants vs 15.5% of delayed cases performed by nonconsultants (*P*=0.017). A favorable trend was noted regarding mortality at 1 year for consultant-led operations vs nonconsultant-led operations (22.1% vs 27.9%, respectively; *P*=0.058).

**Table 4.** Outcomes by Surgeon Experience Level

	Surgeon Experience Level		
Outcome	Nonconsultant,^a^ n=690	Consultant,^b^ n=473	*P* Value
Mortality at 1 year			0.058
No	343 (72.1)	268 (77.9)	
Yes	133 (27.9)	76 (22.1)	
Unknown	214	129	
Acute mortality (<24 hours)			0.2
No	663 (96.2)	461 (97.5)	
Yes	26 (3.8)	12 (2.5)	
Unknown	1	0	
Subacute mortality (1 to 30 days)			0.2
No	512 (91.4)	364 (93.6)	
Yes	48 (8.6)	25 (6.4)	
Unknown	130	84	
Mobility at 1 year			0.2
Improved	40 (11.6)	38 (14.3)	
No change	119 (34.6)	74 (27.8)	
Declined	185 (53.8)	154 (57.9)	
Unknown	346	207	
Change in mobility at 1 year, median [IQR]	–1.00 [–3.00, 0.00]	–1.00 [–3.00, 0.00]	>0.9
Preoperative delay (>48 hours)			0.017
No	583 (84.5)	374 (79.1)	
Yes	107 (15.5)	99 (20.9)	
Surgical duration, hours, median [IQR]	1.30 [1.03, 1.68]	1.03 [0.79, 1.38]	<0.001
Unknown	13	2	

^a^Nonconsultants are surgeons still in training such as fellows, principal house officers, and registrars (residents).

^b^Consultants are attending-level surgeons (senior medical officers and specialists).

Notes: Surgeon experience level was not available for 1 patient. Data are presented as n (%) unless otherwise indicated. *P* values for variables reported as n (%) were determined with the Pearson chi-square test; *P* values for variables reported as median [IQR] were determined with the Mann-Whitney test.

IQR, interquartile range.

### Logistic Regression Analysis

Table 5 presents the logistic regression analyses of clinical outcomes by hospital admission time point and surgeon experience level. Mortality at 1 year was significant in the hospital admission time point analysis, and preoperative delay was significant in the surgeon experience level analysis.

**Table 5.** Logistic Regression Analysis of Clinical Outcomes by Hospital Admission Time Point and Surgeon Experience Level

	Hospital Admission Time Point		
Outcome	Number of Patients	Odds Ratio (95% CI)	*P* Value
Mortality at 1 year	821	1.45 (1.04, 2.01)	0.029
Acute mortality (<24 hours)	1,163	1.27 (0.63, 2.45)	0.5
Subacute mortality (1 to 30 days)	950	1.54 (0.94, 2.49)	0.085
Preoperative delay (>48 hours)	1,164	0.98 (0.71, 1.35)	0.9
	**Surgeon Experience Level**		
**Outcome**	**Number of Patients** ^a^	**Odds Ratio (95% CI)**	***P* Value**
Mortality at 1 year	820	0.73 (0.53, 1.01)	0.058
Acute mortality (<24 hours)	1,162	0.66 (0.32, 1.30)	0.2
Subacute mortality (1 to 30 days)	949	0.73 (0.44, 1.20)	0.2
Preoperative delay (>48 hours)	1,163	0.69 (0.51, 0.94)	0.017

^a^Surgeon experience level was not available for 1 patient.

Note: Binomial logistic regression was performed on each binary clinical outcome to determine odds ratios.

Although Table 4 showed a favorable trend for 1-year mortality for consultant-led vs nonconsultant-led operations, after adjusting for type of operation, consultant status no longer trended toward decreased mortality at 1 year (Table 6).

**Table 6.** Logistic Regression Analysis of 1-Year Mortality by Surgeon Experience Level and Type of Operation

Variable	Number of Patients	Odds Ratio (95% CI)	*P* Value
Surgeon experience level			
Consultant	473	0.76 (0.54, 1.07)	0.12
Type of operation			
Cannulated screws	41	—	—
Dynamic hip screws	86	3.35 (0.82, 22.7)	0.13
Hemiarthroplasty	367	6.61 (1.89, 41.9)	0.012
Intramedullary nail	529	3.99 (1.15, 25.2)	0.064
Total hip arthroplasty	135	1.35 (0.32, 9.19)	0.7

Notes: Significance was determined using multivariate binomial logistic regression. One-year mortality by consultant status was analyzed and adjusted for type of operation to assess for confounding. Because of the low frequency of cannulated screws surgeries, mortality at 1 year could not be assessed. Data were unavailable for 6 patients.

In the analysis of 1-year mortality by type of operation, hemiarthroplasty was associated with increased mortality at 1 year (*P*=0.012) (Table 6). However, no significant associations were seen between operation type and delay to surgery (>48 hours) (Table 7).

**Table 7.** Logistic Regression Analysis of Preoperative Delay by Surgeon Experience Level and Type of Operation

Variable	Number of Patients	Odds Ratio (95% CI)	*P* Value
Surgeon experience level			
Consultant	473	0.72 (0.53, 0.99)	0.043
Type of operation			
Cannulated screws	41	—	—
Dynamic hip screws	86	1.18 (0.48, 2.80)	0.7
Hemiarthroplasty	367	1.68 (0.75, 3.52)	0.2
Intramedullary nail	529	1.77 (0.80, 3.63)	0.14
Total hip arthroplasty	135	1.13 (0.48, 2.53)	0.8

Notes: Significance was determined using a multivariate binomial logistic regression. Preoperative delay by consultant status was analyzed and adjusted for type of operation to assess for confounding. Because of the low frequency of cannulated screws surgeries, preoperative delay could not be assessed. Data were unavailable for 6 patients.

## DISCUSSION

Evidence is clear that a weekend effect exists for a wide variety of emergent surgical procedures, with studies showing an association between weekend admission and higher rates of morbidity and mortality.^12,13,20,27-29^ Controversy regarding the existence of a weekend effect for hip fractures is ongoing. Our retrospective study of 1,164 patients demonstrated that weekend admissions had a significant effect on patient mortality rates at 1 year (30.4% vs 23.2%; *P*=0.029), but no other significant negative outcomes were observed in the study. The weekend cohort had a higher subacute mortality (1 to 30 days) rate than the weekday cohort (9.9% vs 6.7%, respectively), although this difference only trended toward significance (*P*=0.083). We found no significant differences in mobility change from baseline (*P*=0.12) or preoperative delay (*P*=0.9) to help explain the mortality rate.

Weekend admission was associated with a significant, but minimal, difference in surgical duration compared to weekday admission: a <5-minute (0.08 hour) difference between median times (1.15 hours vs 1.23 hours, respectively; *P*<0.001). This difference is unlikely to justify the increased mortality rate, as shorter procedures are typically associated with lower complication rates (wound infection, revision, and death) in joint arthroplasty cases.^30-32^

Hemiarthroplasty was associated with increased 1-year mortality (*P*=0.012), but the fewer hemiarthroplasties performed on weekends than weekdays (29.9% vs 32.4%, respectively) do not explain the increased 1-year mortality rate in the weekend cohort. Furthermore, we found no significant associations between operation type and a >48-hour delay to surgery.

While one could theorize that patients admitted on the weekends have more significant preoperative risk factors than patients admitted on weekdays, we found no association between ASA grades (*P*=0.2) or prior hip fracture (*P*=0.2) and hospital admission time point.

Consultant surgeons performed significantly more of the cases delayed >48 hours (*P*=0.017). This trend could be partially explained by the fact that consultant-level surgeons were performing surgeries on higher risk patients who required more extensive preoperative optimization or on the more difficult cases. However, we found no difference in surgeon experience level between admission time point cohorts (*P*=0.2) and no relationship between hospital admission time point and preoperative delay (*P*=0.9).

While many potential explanations exist for differences in weekend outcomes, no clear cause has been established for hip fractures. In some cases, weekend admission may be associated with sicker patients or more severe injuries, leading to a higher rate of negative outcomes.^33,34^ Conventionally, degree of injury severity is unlikely to be the cause of the hip fracture weekend effect as hip fractures require urgent treatment and patients are unlikely to delay presentation. However, a 2020 study demonstrated that only 66.3% of patients presented within 2 days while some waited until weeks after injury,^35^ but whether this delay is related to a weekend effect is unclear. Another widely accepted hypothesis is that surgeon experience level plays a role in the weekend effect, with the assumption that upper-level surgeons are less likely to perform weekend procedures, thereby leading to poorer outcomes. However, we found no relationship between surgeon experience level and hospital admission time point.

Given the retrospective nature of our study, we were unable to account for potential differences in nursing staff experience and availability that have been shown to impact weekend operations and outcomes in other fields.^36,37^ Similarly, we were not able to account for the availability of services such as physiotherapy, occupational therapy, and geriatric specialists on weekends. Boutera et al suggested changes in discharge and follow-up after weekend admission as potential causes for an increased 2-month mortality.^22^ Asheim et al supported this explanation of possible postoperative factors impacting weekend admission.^38^ In their study, weekend discharge was associated with poorer long-term outcomes, including increased mortality.^38^

Our study has several limitations, including the retrospective nature, the narrow focus on a single institution, and the smaller sample size compared to other studies, making it difficult to account for differences between departmental protocols. In addition, follow-up data at 1 year were limited, likely because the quaternary hospital treats a predominantly rural population, and the patients can be difficult to contact. Future studies that look at longer term outcomes may highlight additional differences between the groups.

## CONCLUSION

Among this fragility hip fracture cohort, weekend admission was associated with increased 1-year mortality. This study provides important evidence that a clinically significant weekend effect cannot be dismissed regarding hip fracture treatments. The need for further evaluation of possible causes for the weekend effect to improve future care is clear.

## Notes

*This article meets the Accreditation Council for Graduate Medical Education and the American Board of Medical Specialties Maintenance of Certification competencies for Patient Care, Medical Knowledge, and Practice-Based Learning and Improvement.*

## References

[1] Johnell O, Kanis JA. An estimate of the worldwide prevalence and disability associated with osteoporotic fractures. Osteoporos Int. 2006;17(12):1726-1733. doi: 10.1007/s00198-006-0172-4 16983459

[2] Cooper C, Campion G, Melton LJ 3rd. Hip fractures in the elderly: a world-wide projection. Osteoporos Int. 1992;2(6):285-289. doi: 10.1007/BF01623184 1421796

[3] Cooper C, Cole ZA, Holroyd CR, Secular trends in the incidence of hip and other osteoporotic fractures. Osteoporos Int. 2011;22(5):1277-1288. doi: 10.1007/s00198-011-1601-6 21461721 PMC3546313

[4] Hip fracture incidence and hospitalisations in Australia 2015-16. Australian Institute of Health and Welfare. Updated August 15, 2023. Accessed November 8, 2024. aihw.gov.au/reports/injury/hip-fracture-incidence-in-australia-2015-16/summary

[5] Johansen A, Tsang C, Boulton C, Wakeman R, Moppett I. Understanding mortality rates after hip fracture repair using ASA physical status in the National Hip Fracture Database. Anaesthesia. 2017;72(8):961-966. doi: 10.1111/anae.13908 28585391

[6] Moore DP, Quinlan W. Mortality and morbidity associated with hip fractures. Ir J Med Sci. 1989;158(2):40-42. doi: 10.1007/BF02942060 2745031

[7] Bentler SE, Liu L, Obrizan M, The aftermath of hip fracture: discharge placement, functional status change, and mortality. Am J Epidemiol. 2009;170(10):1290-1299. doi: 10.1093/aje/kwp266 19808632 PMC2781759

[8] Newcastle survey of deaths in early childhood 1974/76, with special reference to sudden unexpected deaths. Working party for early childhood deaths in Newcastle. Arch Dis Child. 1977;52(11):828-835. doi: 10.1136/adc.52.11.828 596921 PMC1544816

[9] Kostis WJ, Demissie K, Marcella SW, Weekend versus weekday admission and mortality from myocardial infarction. N Engl J Med. 2007;356(11):1099-1109. doi: 10.1056/NEJMoa063355 17360988

[10] Jang KM, Jang JS. Weekend admission and mortality in patients with traumatic brain injury: a meta-analysis. Korean J Neurotrauma. 2023;19(4):422-433. doi: 10.13004/kjnt.2023.19.e61 38222828 PMC10782108

[11] O'Leary JD, Wunsch H, Leo AM, Levin D, Siddiqui A, Crawford MW. Hospital admission on weekends for patients who have surgery and 30-day mortality in Ontario, Canada: a matched cohort study. PLoS Med. 2019;16(1):e1002731. doi: 10.1371/journal.pmed.1002731 30695035 PMC6350956

[12] Bell CM, Redelmeier DA. Mortality among patients admitted to hospitals on weekends as compared with weekdays. N Engl J Med. 2001;345(9):663-668. doi: 10.1056/NEJMsa003376 11547721

[13] Roberts SE, Thorne K, Akbari A, Samuel DG, Williams JG. Weekend emergency admissions and mortality in England and Wales. Lancet. 2015;385(9980):1829. doi: 10.1016/S0140-6736(15)60580-3 25957452

[14] Daugaard CL, Jørgensen HL, Riis T, Lauritzen JB, Duus BR, van der Mark S. Is mortality after hip fracture associated with surgical delay or admission during weekends and public holidays? A retrospective study of 38,020 patients. Acta Orthop. 2012;83(6):609-613. doi: 10.3109/17453674.2012.747926 23140106 PMC3555458

[15] Smith T, Pelpola K, Ball M, Ong A, Myint PK. Pre-operative indicators for mortality following hip fracture surgery: a systematic review and meta-analysis. Age Ageing. 2014;43(4):464-471. doi: 10.1093/ageing/afu065 24895018

[16] Moran CG, Wenn RT, Sikand M, Taylor AM. Early mortality after hip fracture: is delay before surgery important? J Bone Joint Surg Am. 2005;87(3):483-489. doi: 10.2106/JBJS.D.01796 15741611

[17] Sayers A, Whitehouse MR, Berstock JR, Harding KA, Kelly MB, Chesser TJ. The association between the day of the week of milestones in the care pathway of patients with hip fracture and 30-day mortality: findings from a prospective national registry – The National Hip Fracture Database of England and Wales. BMC Med. 2017;15(1):62. doi: 10.1186/s12916-017-0825-5 28343451 PMC5367007

[18] Ricci WM, Brandt A, McAndrew C, Gardner MJ. Factors affecting delay to surgery and length of stay for patients with hip fracture. J Orthop Trauma. 2015;29(3):e109-e114. doi: 10.1097/BOT.0000000000000221 25186844 PMC4339640

[19] Authen AL, Dybvik E, Furnes O, Gjertsen JE. Surgeon's experience level and risk of reoperation after hip fracture surgery: an observational study on 30,945 patients in the Norwegian Hip Fracture Register 2011-2015. Acta Orthop. 2018;89(5):496-502. doi: 10.1080/17453674.2018.1481588 29860911 PMC6202762

[20] Mathews JA, Vindlacheruvu M, Khanduja V. Is there a weekend effect in hip fracture patients presenting to a United Kingdom teaching hospital? World J Orthop. 2016;7(10):678-686. doi: 10.5312/wjo.v7.i10.678 27795950 PMC5065675

[21] Sheikh HQ, Aqil A, Hossain FS, Kapoor H. There is no weekend effect in hip fracture surgery–a comprehensive analysis of outcomes. Surgeon. 2018;16(5):259-264. doi: 10.1016/j.surge.2017.11.001 29191435

[22] Boutera A, Dybvik E, Hallan G, Gjertsen JE. Is there a weekend effect after hip fracture surgery? A study of 74,410 hip fractures reported to the Norwegian Hip Fracture Register. Acta Orthop. 2020;91(1):63-68. doi: 10.1080/17453674.2019.1683945 31663395 PMC7008236

[23] Hatchimonji JS, Kaufman EJ, Sharoky CE, Ma LW, Holena DN. A 'weekend effect' in operative emergency general surgery. Am J Surg. 2020;220(1):237-239. doi: 10.1016/j.amjsurg.2019.11.024 31744597

[24] Boylan MR, Riesgo AM, Paulino CB, Tejwani NC. Day of admission is associated with variation in geriatric hip fracture care. J Am Acad Orthop Surg. 2019;27(1):e33-e40. doi: 10.5435/JAAOS-D-17-00143 30247307

[25] Downey C, Kelly M, Quinlan JF. Changing trends in the mortality rate at 1-year post hip fracture – a systematic review. World J Orthop. 2019;10(3):166-175. doi: 10.5312/wjo.v10.i3.166 30918799 PMC6428998

[26] Parker MJ, Palmer CR. A new mobility score for predicting mortality after hip fracture. J Bone Joint Surg Br. 1993;75(5):797-798. doi: 10.1302/0301-620X.75B5.8376443 8376443

[27] Shih PC, Liu SJ, Li ST, Chiu AC, Wang PC, Liu LY. Weekend effect in upper gastrointestinal bleeding: a systematic review and meta-analysis. PeerJ. 2018;6:e4248. doi: 10.7717/peerj.4248 29340247 PMC5768163

[28] Smith SA, Yamamoto JM, Roberts DJ, Weekend surgical care and postoperative mortality: a systematic review and meta-analysis of cohort studies. Med Care. 2018;56(2):121-129. doi: 10.1097/MLR.0000000000000860 29251716 PMC5770102

[29] Beller JP, Chancellor WZ, Mehaffey JH, Outcomes of non-elective coronary artery bypass grafting performed on weekends. Eur J Cardiothorac Surg. 2020;57(6):1130-1136. doi: 10.1093/ejcts/ezz379 31986194 PMC7239602

[30] Belmont PJ Jr, Goodman GP, Waterman BR, Bader JO, Schoenfeld AJ. Thirty-day postoperative complications and mortality following total knee arthroplasty: incidence and risk factors among a national sample of 15,321 patients. J Bone Joint Surg Am. 2014;96(1):20-26. doi: 10.2106/JBJS.M.00018 24382720

[31] O'Malley NT, Fleming FJ, Gunzler DD, Messing SP, Kates SL. Factors independently associated with complications and length of stay after hip arthroplasty: analysis of the National Surgical Quality Improvement Program. J Arthroplasty. 2012;27(10):1832-1837. doi: 10.1016/j.arth.2012.04.025 22810006

[32] Ong KL, Lau E, Manley M, Kurtz SM. Effect of procedure duration on total hip arthroplasty and total knee arthroplasty survivorship in the United States Medicare population. J Arthroplasty. 2008;23(6 Suppl 1):127-132. doi: 10.1016/j.arth.2008.04.022 18555641

[33] Han L, Meacock R, Anselmi L, Variations in mortality across the week following emergency admission to hospital: linked retrospective observational analyses of hospital episode data in England, 2004/5 to 2013/14. Southampton (UK): NIHR Journals Library; November 2017. 29172363

[34] Sun J, Girling AJ, Aldridge C, Sicker patients account for the weekend mortality effect among adult emergency admissions to a large hospital trust. BMJ Qual Saf. 2019;28(3):223-230. doi: 10.1136/bmjqs-2018-008219 PMC6560459 30301873

[35] He W, You YY, Sun K, Admission delay is associated with worse surgical outcomes for elderly hip fracture patients: a retrospective observational study. World J Emerg Med. 2020;11(1):27-32. 31893000 10.5847/wjem.j.1920-8642.2020.01.004 PMC6885583

[36] Cram P, Hillis SL, Barnett M, Rosenthal GE. Effects of weekend admission and hospital teaching status on in-hospital mortality. Am J Med. 2004;117(3):151-157. doi: 10.1016/j.amjmed.2004.02.035 15276592

[37] Pauls LA, Johnson-Paben R, McGready J, Murphy JD, Pronovost PJ, Wu CL. The weekend effect in hospitalized patients: a meta-analysis. J Hosp Med. 2017;12(9):760-766. doi: 10.12788/jhm.2815 28914284

[38] Asheim A, Nilsen SM, Toch-Marquardt M, Anthun KS, Johnsen LG, Bjørngaard JH. Time of admission and mortality after hip fracture: a detailed look at the weekend effect in a nationwide study of 55,211 hip fracture patients in Norway. Acta Orthop. 2018;89(6):610-614. doi: 10.1080/17453674.2018.1533769 30398406 PMC6319186

